# Antibiotic use during coronavirus disease 2019 intensive care unit shape multidrug resistance bacteriuria: A Swedish longitudinal prospective study

**DOI:** 10.3389/fmed.2023.1087446

**Published:** 2023-02-07

**Authors:** Philip A. Karlsson, Julia Pärssinen, Erik A. Danielsson, Nikos Fatsis-Kavalopoulos, Robert Frithiof, Michael Hultström, Miklos Lipcsey, Josef D. Järhult, Helen Wang

**Affiliations:** ^1^Department of Medical Biochemistry and Microbiology, Uppsala University, Uppsala, Sweden; ^2^Department of Surgical Sciences, Anesthesiology and Intensive Care Medicine, Uppsala University, Uppsala, Sweden; ^3^Department of Medical Cell Biology, Integrative Physiology, Uppsala University, Uppsala, Sweden; ^4^Hedenstierna Laboratory, CIRRUS, Department of Surgical Sciences, Anesthesiology and Intensive Care Medicine, Uppsala University, Uppsala, Sweden; ^5^Department of Medical Sciences, Zoonosis Science Center, Uppsala University, Uppsala, Sweden

**Keywords:** UTI, ICU–intensive care unit, COVID-19, MDR–(multidrug resistance), AMR, antibiotic treatment, catheters

## Abstract

**Objectives:**

High frequency of antimicrobial prescription and the nature of prolonged illness in COVID-19 increases risk for complicated bacteriuria and antibiotic resistance. We investigated risk factors for bacteriuria in the ICU and the correlation between antibiotic treatment and persistent bacteria.

**Methods:**

We conducted a prospective longitudinal study with urine from indwelling catheters of 101 ICU patients from Uppsala University Hospital, Sweden. Samples were screened and isolates confirmed with MALDI-TOF and whole genome sequencing. Isolates were analyzed for AMR using broth microdilution. Clinical data were assessed for correlation with bacteriuria.

**Results:**

Length of stay linearly correlated with bacteriuria (R^2^ = 0.99, *p* ≤ 0.0001). 90% of patients received antibiotics, primarily the beta-lactams (76%) cefotaxime, piperacillin-tazobactam, and meropenem. We found high prevalence of *Enterococcus* (42%) being associated with increased cefotaxime prescription. Antibiotic-susceptible *E. coli* were found to cause bacteriuria despite concurrent antibiotic treatment when found in co-culture with *Enterococcus*.

**Conclusion:**

Longer stays in ICUs increase the risk for bacteriuria in a predictable manner. Likely, high use of cefotaxime drives *Enterococcus* prevalence, which in turn permit co-colonizing Gram-negative bacteria. Our results suggest biofilms in urinary catheters as a reservoir of pathogenic bacteria with the potential to develop and disseminate AMR.

## Introduction

Urinary tract infections (UTIs) is a major reason for healthcare-associated infections (HAIs) globally (1). In Sweden, UTIs are recognized as the principle cause of HAIs (2). Intensive care unit (ICU) treatment is one of few recognized indications for catheterization, and indwelling catheters are the main source of nosocomial UTIs. Severely ill COVID-19 patients are principally treated in ICUs with associated catheterization, giving this group of patients risk of complicated UTIs, defined by high rates of treatment failure (3). An additional distress is that clinical symptoms of nosocomial UTIs might be concealed by COVID-19-associated damage, potentially increasing the risk of prolonged UTI-related impairment. Alongside systemically used immunosuppressive treatment in this patient group, meta-analyses have revealed that 86% of COVID-19 ICU patients receive antibiotics (4). This raises concerns for atypical infections and multidrug resistance (MDR). Still, data and correlation analysis on antibiotic use, bacterial prevalence, and antimicrobial resistance (AMR) are limited. There is no longitudinal study on AMR development in the COVID-19 ICU cohort.

This prospective study was based on a cohort of COVID-19 patients from the ICU in Uppsala University Hospital, Sweden. We performed longitudinal screening for bacteriuria as well as collected underlying medical, diagnostic, and treatment data. Urinary isolates were consecutively tested for AMR. This study had three aims: to investigate how bacteriuria correlate with length of stay (LOS) and additional clinical variables; how specific treatment correlate to specific colonization patterns; and to explore whether antibiotic-susceptible bacteria persist during treatment. To our knowledge, this is the first report to show how longitudinal antibiotic treatment correlates with bacterial prevalence and AMR in ICU-patients.

## Materials and methods

### Sample collection and storage

Clinical data were recorded daily and included age, sex, LOS, simplified acute physiology score 3 (SAPS3) at arrival, diabetes, hospitalization outcome, immunosuppressive treatment, antibiotic treatment, and findings from clinical microbiology (clinical routine samples, regular monitoring). All admitted patients received transurethral catheterization in a closed system as part of clinical practice. Urine study samples were collected every Monday, Wednesday and Friday (separate from regular monitoring) aseptically from the catheter into sterile vacutainer tubes and transported cold. Urine study samples were processed within 2 h of collection by aliquoting from vacutainers into cryovials with 10% dimethyl sulfoxide (DMSO) for storage in –80°C. Bacterial isolates from routine samples were not assessed in this study. All samples and data were collected from patients fulfilling the inclusion criteria in any of the intensive care unit facilities at Uppsala University Hospital, Sweden.

### Species identification and cultivation

In short, urine was plated onto Brilliance™ UTI Clarity™ agar. Significant growth was considered >10^3^ CFU/ml (>10^5^ for *Staphylococcus epidermidis*) based on national guidelines for UTIs (Supplementary Table 1: includes ECDC UTI comparison). It was in this study assumed that assessment of UTI symptomatology for the cohort might have been compromised. As clinical symptoms could not be assessed, samples are not described in terms of UTIs but instead as bacteriuria/non-bacteriuria. Species were identified using MALDI-TOF and saved frozen in Brain Heart Infusion (BHI, 10% DMSO). Species confirmation and clonality control was performed for a subset of strains with whole-genome sequencing analysis. All cultivation was carried out at 37°C. For a full description, see Supplementary material.

### Minimum inhibitory concentration testing

Minimum inhibitory concentration (MIC) tests were performed by the European Committee on Antimicrobial Susceptibility Testing (EUCAST) BMD method (V12.0) (5). Experiments were run in biological duplicates in 96-well microtiter plates and bacterial suspensions of 0.5 McFarland standard units were added to each well. Positive (no antibiotic) and negative controls (no bacteria) were added to each plate. Information about procedures, controls and antibiotics are found in Supplementary Table 2. The classification of MDR was based on proposed standard definitions, which in short specifies MDR as resistance against minimum three different classes of clinically relevant antibiotics (6).

### Data processing and statistical analysis

All analysis was performed in GraphPad Prism v9. In discrete and ratiometric parameters, correlations were investigated with chi-square tests unless otherwise specified. In continuous parameters, correlations were investigated using the Spearman correlation tests, and deviations in means with two-tailed *t*-tests. Significance for linear regression was assessed with the likelihood ratio test and the Wald test, and Gaussian distribution (normality) was measured using the D’Agostino-Pearson normality test. A significant difference was identified at *p*-values smaller than 0.05 with * denoting <0.05, ^**^<0.01, ^***^<0.001, and ^****^<0.0001. Cross-correlation was controlled for death, LOS, SAPS3, and age.

## Results

**FIGURE 1:**
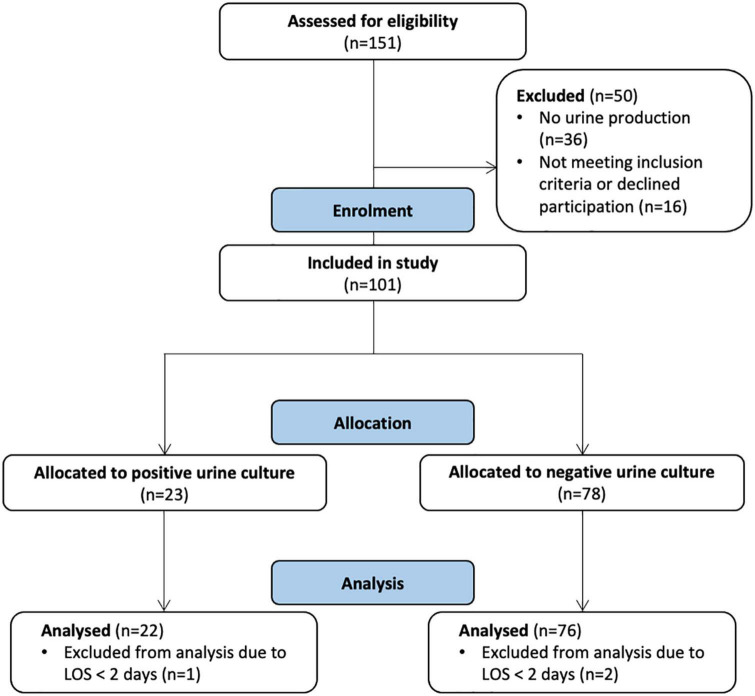
CONsolidated Standards of Reporting Trials (CONSORT) flow diagram for the progress of enrollment and allocation based on bacteriuria (urine study samples).

Between Jun 5th, 2020, and Feb 17th, 2021, there were 21,130 recorded COVID-19 cases in Uppsala County, resulting in 151 patients being treated in intensive care. Out of the 151 patients screened, 101 were enrolled in the study. Three patients with a LOS in the ICU of less than 2 days were excluded from the present analysis (Figure 1).

Age and SAPS3 were normally distributed (D’Agostino-Pearson, ns) while LOS was not (^****^) (Supplementary Figures 1A–C). Participants had a mean age of 65 [standard deviation (SD): 12.80] and a SAPS3 on arrival of 55 (SD: 9.90). Analysis of frequency distribution (automatic 5 days binning) of LOS identified two separate groups with a LOS of longer or shorter than 30 days (Supplementary Figure 1C). LOS was divided into groups of shorter (*n* = 84, x̄: 10.56, SD: 6.56) and longer (*n* = 13, x̄: 40.85, SD: 7.3) stay. Most participants were men (74%) (Table 1). Diabetes was an underlying disease in 36% of the patients and the overall cohort mortality was 18.4% with no significant difference between bacteriuric and non-bacteriuric patients. 89% (*n* = 92) received minimum one immunosuppressant, most commonly dexamethasone (69 patients). 90% (*n* = 90) received at least one antibiotic. Clinical routine samples were positive 78 times across 44 patients (45% of cohort) and our longitudinal urine screen identified 34 potential clones (70 isolates) across 22 patients (23% of cohort). As expected, having any positive clinical routine sample significantly increased the relative risk (RR) of bacteriuria (RR: 3.15^**^), similarly to not having received any antibiotic (RR: 2.65*) (Table 1). Neither sex, diabetes or immunosuppressive treatment increased the risk of bacteriuria.

**TABLE 1:** Characteristics of enrolled coronavirus disease 2019 (COVID-19) intensive care unit (ICU) patients.

Parameter	Bacteriuria	No bacteriuria	Total	Relative risk	95% CI	*P*-value	Significance
**Sex**	22	76	98	0.96	0.45–2.23	0.93	ns
Male	16	56	72	–	–	–	–
Female	6	20	26	–	–	–	–
**Diabetes**	22	76	98	0.68	0.29–1.49	0.35	ns
Yes	6	29	35	–	–	–	–
No	16	47	63	–	–	–	–
**Immunosuppressive**	22	67	89	0.80	0.35–2.37	0.68	ns
Yes	19	60	79	–	–	–	–
No	3	7	10	–	–	–	–
**Deceased (30 days)**	22	76	98	0.44	0.12–1.42	0.20	ns
Yes	2	16	18	–	–	–	–
No	20	60	80	–	–	–	–
**Bacterial findings in clinical routine samples**	22	76	98	3.15	1.40–7.28	0.004	**
Yes	16	29	44	–	–	–	–
No	8	46	54	–	–	–	–
**Bacteriuria from clinical routine samples**	22	76	98	5.97	3.22–10.6	<0.0001	****
Yes	10	2	12	–	–	–	–
No	12	74	86	–	–	–	–
**Antibiotic treatment**	22	68	90	2.65	1.16–4.93	0.02	*
No	5	4	9	–	–	–	–
Yes	17	64	81	–	–	–	–

*P < 0.05, **P < 0.01, and ****P < 0.0001.

**FIGURE 2:**
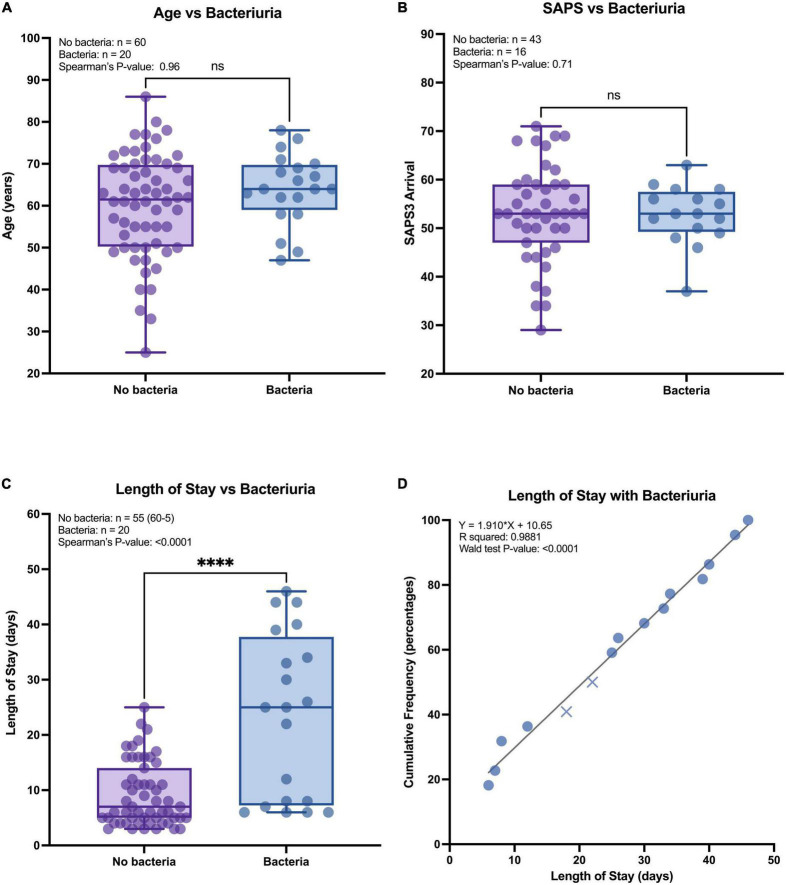
Continuous parameters and risk of bacteriuria. **(A–C)** All patients were grouped by urine colonization, no (purple) and yes (blue), compared against three continuous parameters. The comparison was measured with a two-tailed *t*-test (illustration) and Spearman’s rank correlation (top left text). ****P < 0.0001. **(D)** Patients with urine study samples were sorted by cumulative frequency against the length of stay (LOS) and tested against a linear regression model (significance measured with the likelihood test and Wald test). Pattern indicate patients that survived (circle) and patients that died (cross).

No correlation was found between bacteriuria and age or SAPS3, as seen in Figures 2A, B (Spearman’s rank correlation), and there were no significant deviations in mean age or SAPS3 of patients with bacteriuria (post-hoc, two-tailed *t*-test). LOS was significantly different when comparing patients with and without bacteriuria (Spearman/*t*-test^***^) (Figure 2C). When comparing shorter and longer LOS, a two-sided chi-square test demonstrated a significant correlation with an increased RR of 2.17 [95% confidence interval (CI 95%): 1.78–4.74] for LOS above 30 days (chi-squared^***^) (Supplementary Figure 1C). Cumulative frequency of bacteriuria over LOS showed a linear relationship (R^2^: 0.99, likelihood/Wald test^****^) with a slope of 1.91 (CI 95%: 1.78–2.04) (Figure 2D). This relationship indicates that bacteriuria occurred systematically in this setting, and not only as a result of increasing probability over time. Given the 23%-point prevalence in the cohort, the RR of developing bacteriuria increased by 0.44% for each day spent in the ICU. Age, LOS, SAPS3, and death were analyzed for cross-correlation, but only low or non-significant correlation could be observed (Supplementary Figure 1D).

**FIGURE 3:**
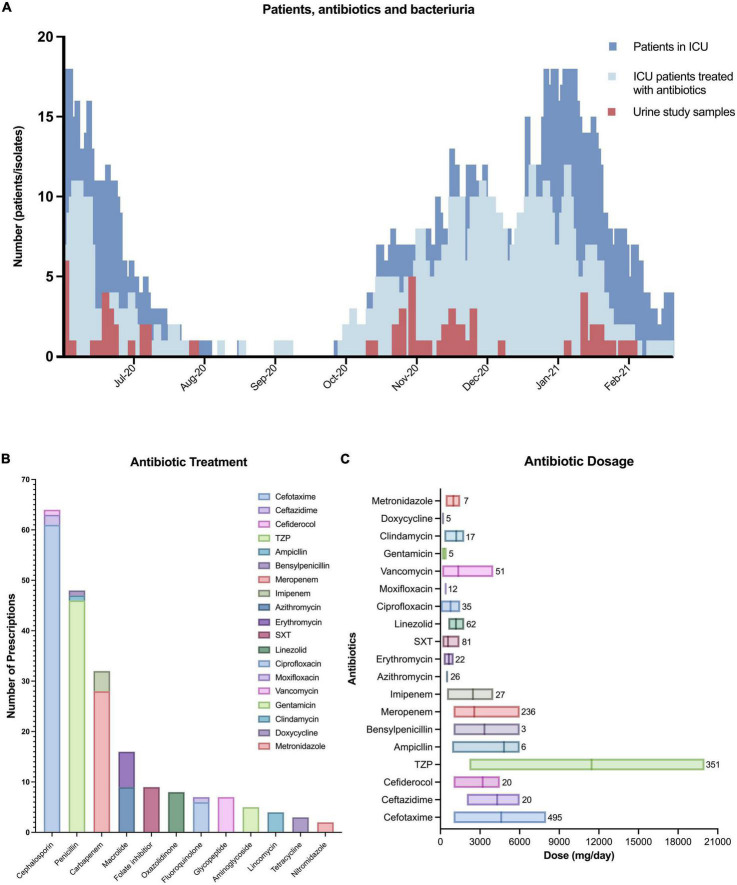
Antibiotic treatment. **(A)** Epidemiological overview of all patients admitted to the Uppsala university hospital intensive care unit (dark blue) according to the Swedish Intensive Care Registry (SIR), the number of ICU patients in our cohort receiving at least one antibiotic (bright blue), and the number of urine study samples identified (red). **(B)** The number of prescribed antibiotics where each antibiotic is counted maximum once per patient. TZP, piperacillin-tazobactam; SXT, trimethoprim-sulfamethoxazole. **(C)** The dose of antibiotic prescriptions with the internal line marking the mean and the box number indicates the total number of prescription events.

Eighty-one individuals received a total of 253 antibiotic prescriptions (*n* = 90), not including multiple prescriptions of the same drug within a patient (Figures 3A, B). The most common class was β-lactams (*n* = 192, 76%), comprising cephalosporins (*n* = 64), penicillins (*n* = 49), and carbapenems (*n* = 32). Nearly all respective treatment consisted of cefotaxime (*n* = 61), piperacillin-tazobactam (TZP, *n* = 47) or meropenem (*n* = 28), and together these three drugs represented 54% of all prescriptions. TZP was prescribed with large dose variation between patients (Figure 3C). Following β-lactams, the most common classes were macrolides, trimethoprim-sulfamethoxazole and linezolid.

**FIGURE 4:**
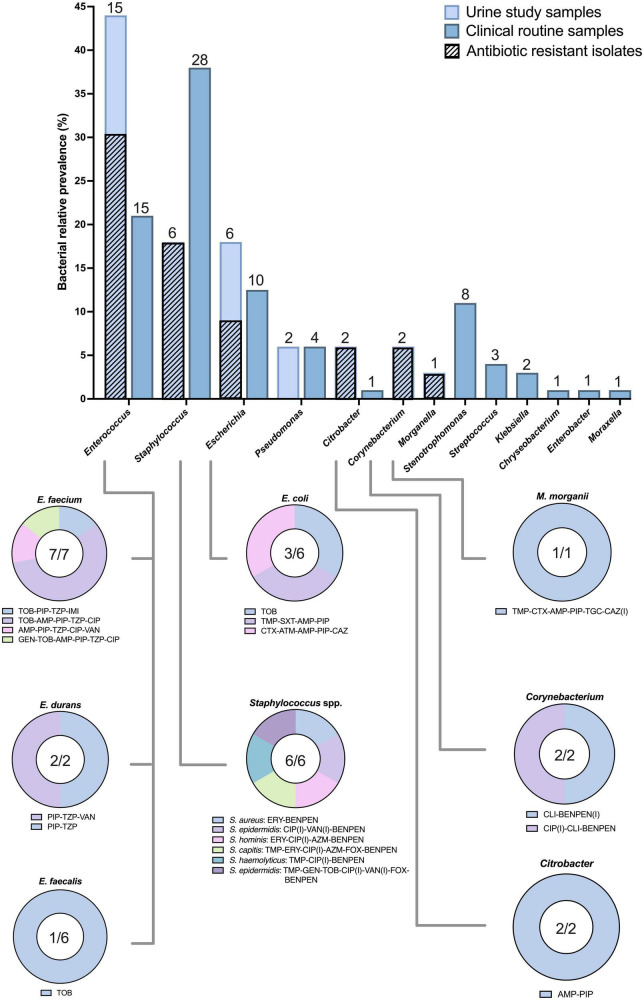
Relative prevalence and antibiotic resistant isolates. The relative prevalence of identified bacteria in urine study samples (bright blue) and any positive clinical routine sample (dark blue). The number above the bar shows the number of bacteria where each species is counted maximum once per patient. Diagonal patterns (bright blue bars) indicate resistance against at least one tested antibiotic. The lower panel illustrates identified resistant phenotypes, with the number within the circle graph showing the number of resistant bacteria in relation to the total number identified. Abbreviations for antibiotics can be found in Supplementary Table 1.

Clinical routine samples (all sampling sites) were registered as standard procedure during regular clinical monitoring, while urine study samples (longitudinal urine) were collected separately from clinical routine. In clinical routine samples, 45% (*n* = 98) were identified with 74 positive bacterial findings (multiple per patient). These were mainly isolated from the respiratory tract (*n* = 41, 54%) and blood (*n* = 21, 29%), followed by urine (*n* = 12, 15%) and wound (*n* = 2, 3%). Most cultures belonged to the genus *Staphylococcus* (*n* = 28, 38%), followed by *Enterococcus* (*n* = 15, 21%), *Escherichia* (*n* = 10, 13%) and *Stenotrophomonas* (*n* = 8, 11%) (Figure 4). The majority of findings were Gram-positive (*n* = 45, 63%).

In our study; 70 urine study sample isolates were identified from 22 patients. For better comparison with (mostly cross-sectional) clinical routine samples, each species was only counted once per patient (34 potential clones). The most frequent genus was *Enterococcus* (*n* = 15, 42%), followed by *Staphylococcus* (*n* = 6, 18%) and *Escherichia* (*n* = 6, 18%). Similar to clinical routine samples, the urine study samples were mainly Gram-positive (*n* = 22, 67%) (Figure 4). We re-identified 10/12 of clinical routine urine samples. Our criteria classified significant bacterial growth as a CFU larger than 10^3^ (10^5^ for *S. epidermidis*), and antibiotic resistance as an MIC at least 2-fold above the clinical breakpoint (EUCAST) for minimum one of the isolates per patient (Supplementary Tables 1, 2). The average day for the first isolate to appear was 15.68 days (SD: 12.35), which tended to be smaller for *Staphylococcus* (*n* = 6, x̄: 8.67, SD: 11.72) and larger for *Enterococcus* (*n* = 15, x̄: 19.60, SD: 10.87). Mean of *E. coli* first appearance computes similar to *Staphylococcus* (*n* = 6, x̄: 10.33, SD: 9.22), but taking distribution into account, *Staphylococcus* generally appeared earlier in colonization than *E. coli*, while *Enterococcus* appeared more constant throughout the days in the ICU (Supplementary Table 1). Multiple patients carried bacteria from the WHO global priority list of AMR pathogens, including two third-generation cephalosporin-resistant *Enterobacteriaceae* (3GCRE, critical) (Figure 4): one MDR *M. morganii* and one ESBL-producing MDR *E. coli*. Importantly, this *E. coli* was the only *Escherichia* isolate successfully colonizing a patient without the presence of a Gram-positive co-colonizer. One *E. faecalis* and all but one *E. faecium* presented high-level tobramycin resistance (HLTR). One *E. faecium* and one *E. durans* were identified with probable vancomycin resistance (Supplementary Figure 2 and Supplementary Table 2). In total, three *E. faecium* strains classified as MDR. A proportion of *Enterococcus* isolates surprisingly demonstrated higher piperacillin (PIP) MIC when adding tazobactam in combination. These results were confirmed with E-tests and 24-hour bioscreen growth experiments (for a subset). One *E. faecium* demonstrated a deviation from the EUCAST screen recommendations with resistance against PIP while being susceptible to ampicillin. This strain was additionally resistant to imipenem and results were confirmed with E-tests (Supplementary Figure 2). No antibiotic resistant *Pseudomonas* was identified, but all isolates showed the typical phenotype “susceptible increased exposure” against aztreonam, ciprofloxacin and ceftazidime. All *Staphylococcus* were resistant against benzylpenicillin (used as penicillinase screen in *S. aureus*), but no MRSA was identified (inferred from cefoxitin screen). One *S. hominis* and one *S. epidermidis* were resistant against cefoxitin, indicating methicillin and complete β-lactam-β-lactamase inhibitor resistance. Troublingly, the same *S. epidermidis*, along with a second isolate of the same species, demonstrated “potential vancomycin impaired clinical response” (VAN, Supplementary Table 1). The same MDR *S. epidermidis* co-colonized the patient with susceptible *E. coli*. Apart from *S. epidermidis*, an additional MDR was classified in *S. capitis*.

**FIGURE 5:**
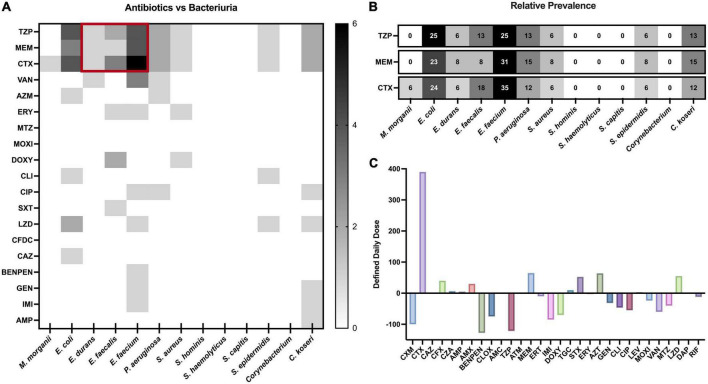
Relationship between antibiotic use and bacteriuria. **(A)** Heatmap correlation between bacteriuria and antibiotic treatment illustrated in numbers. Abbreviations for antibiotics can be found in Supplementary Table 1. **(B)** Correlation heatmap showing the proportion within a species that was exposed to a specific antibiotic. Analysis pool based on that the patient received at least one antibiotic (any) and was colonized by minimum one species (any). Numbers represent the percentage of isolates exposed to the given antibiotic (TZP/MEM/CTX). The percentage for any given antibiotic can be above 100% as isolates from different species were occasionally co-colonizing during the same treatment. **(C)** The difference in antibiotic prescription in the Uppsala ICU from 2019 to 2020 (pre-pandemic to pandemic) given in defined daily dose. Positive values indicate prescription increase while negative values indicate prescription decrease.

Given the high prevalence of *Enterococcus* (Figure 4), and the high *Enterococcus* tolerance against the most prescribed treatment (Figure 3A), we decided to investigate the correlation between antibiotic prescription and bacteriuria. Treatment with MEM or CTX was found to correlate with *E. faecium* colonization (Figure 5A). To account for possible biases in prescription and isolate number, we calculated relative prevalence for every strain against those three antibiotics (isolates divided by number of prescriptions). The relative prevalence for *E. faecium* was confirmed again to be significantly higher during MEM and CTX than other strains (chi square test, Figure 5B). There was a clear increase in usage of CTX and MEM during the COVID pandemic when comparing to the pre-pandemic (Figure 5C) (Swedish eHealth Agency and Strama: prescription data in Uppsala County, Inpatient Care, ICU).

**FIGURE 6:**
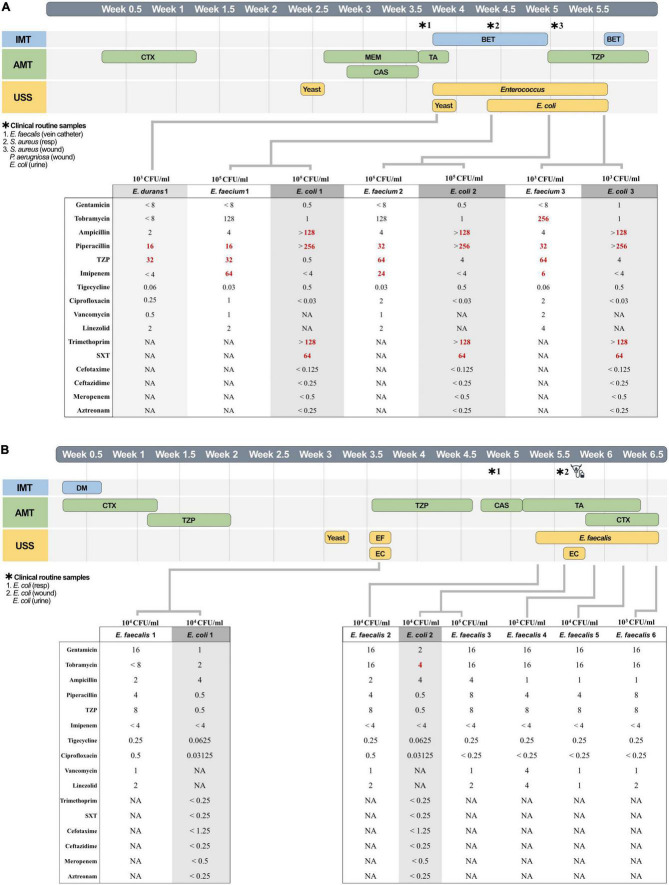
Individual patient timelines. *Clinical routine samples. Timeline of patient A **(A)** and patient B **(B)** at the intensive care unit stays is illustrated in half-weeks. The timeline shows immunomodulatory treatment (IMT, blue), with betamethasone (BET) and dexamethasone (DM), antimicrobial treatment (AMT, green) with caspofungin (CAS), triazole (TA), cefotaxime (CTX), meropenem (MEM), and piperacillin-tazobactam (TZP), and urine study samples (USS, yellow). Stars above the timeline indicate clinical routine samples, and the catheter symbol indicates a change of urinary catheter. The table under the timeline shows the identified minimum inhibitory concentration, with resistance according to The European Committee on Antimicrobial Susceptibility Testing (EUCAST) marked in bold red. In figure **(B)** *E. faecalis* and *E. coli* have been abbreviated EF and EC, respectively.

Investigating *Enterococcus* colonization further, we quantified co-colonization events. We observed that all but one MDR *E. coli* co-colonized with Gram-positive bacteria, four out of five with *Enterococcus*. Only one of these enterococcal co-colonizations occurred with antibiotic-resistant *E. faecium*, while the remaining occurred with antibiotic-susceptible *E. faecalis* (Supplementary Figure 3). To better understand this association between antibiotic prescription and *Enterococcus-Escherichia* colonization over time, we constructed two patient-specific timelines of the patient with resistant *E. faecium* and a patient with susceptible *E. faecalis* (Figure 6).

Figure 6A illustrates patient A who stayed 34 days in the ICU and received early administration of cefotaxime and meropenem. Four days after meropenem, betamethasone administration started, and we identified 10^3^ CFU/ml of *E. durans* in urine. Three days later, *E. durans* was replaced with 10^5^ CFU/ml of *E. faecium* (clonal, novel ST127) and Enteroaggregative *E. coli* (EAEC clonal, ST69). The patient was prescribed TZP against which *E. faecium* was *in vitro* resistant. *E. coli* showed resistance against only PIP but remained during TZP treatment. *E. faecium* and *E. coli* both demonstrated a 2-fold MIC increase against PIP and TZP during active treatment (8-fold increase for TZP in *E. coli*). The MIC for TZP measured in *E. faecium* was consistently twice as high compared to PIP, as previously noted. *E. faecium* were ampicillin-susceptible PIP/TZP/IMI-resistant, despite ampicillin being used by EUCAST for inferred resistance against PIP (Supplementary Table 2). The increase seen for tobramycin marks a change from non-HLTR to HLTR phenotype. To verify the results of ampicillin, imipenem PIP/TZP and tobramycin, BMDs were rerun in conjunction with E-tests, confirming these observations. The tobramycin phenotype indicated heteroresistance when confirmed with E-tests by growth of individual colonies within the zone of clearance.

Figure 6B illustrates patient B who stayed 40 days and received early cefotaxime and TZP treatment, including one day with both drugs simultaneously. Three weeks into intensive care, we identified 10^4^ CFU/ml of *E. faecalis* and Uropathogenic *E. coli* (UPEC). Uncorrelated to our findings, the patient was restarted on TZP treatment that same day, suggestively suppressing colonization in agreement with *in vitro* susceptibility. Interestingly, *E. faecalis* (clone, ST16) re-emerged at 10^4^ CFU/ml soon after stopping treatment, followed 2 days later by the same clonal UPEC (ST10309). The patient received a change of urinary catheter and an administration of cefotaxime. Following that intervention, *E. coli* was no longer found while cephalosporin-tolerant *E. faecalis* remained at a lower concentration (10^2^ CFU/ml) that fluctuated over the last week of intensive care. The second appearance of the strains came with higher MICs for aminoglycosides, including an above clinical breakpoint level for *E. coli*.

## Discussion

To our knowledge, this is the first longitudinal report of bacteriuria and antimicrobial resistance in COVID-19 ICU patients, and the first large-scale epidemiological surveillance of bacteriuria in a Swedish ICU. This study is also the first to show how antibiotic treatment correlates with prevalence of *Enterococcus*, and how co-colonizers can behave in patients *via* patient timelines.

Twenty-three percent of patients experienced bacteriuria with an increased risk of 2.17 when staying more than 30 days. Bacteriuria occurred more frequently in patients surviving intensive care, most likely due to survivors’ bias, with increased risk of colonization by longer LOS. Bacteriuria against LOS showed a linear regression, implying a systematic and potentially preventable occurrence in ICU practice (Figure 2D). The estimated daily risk in our study is lower (0.42%) than previous reports, ranging between 2% and 6% (7–9). A partial explanation to this difference might come from that 90% of our patients received antibiotic treatment, but other reasons include differences in classification of bacteriuria. The Uppsala ICU averaged 2.8 different antibiotics per patient (253/90), similar to the earliest reports of COVID-19 from Wuhan (4, 10). 71% (64/90) of patients received third-generation cephalosporins and 36% (32/90) carbapenems (Figure 3B), both recognized as broad spectrum antibiotics against Gram-negative bacteria. While not receiving antibiotics significantly correlated with bacteriuria (Table 1), our study also illustrates that most bacteria had resistance against at least one tested antibiotic, and that multiple bacteria classified as MDR (Figure 4). Discrepancies in reporting and definitions of resistance remains a concern for comparison of global AMR data (11).

In 45% of our patients, bacteria were detected in clinical routine samples (Table 1). These outcomes are approximately twice as high as HAIs reported from other COVID-19 ICU cohorts, although reliable and comparable data are scarce (12, 13). *Enterococcus* spp. and *Staphylococcus* spp. were most prevalent in both urine study samples and clinical routine samples (Figure 4). The high number of *Enterococcus* is surprising. A large study from the US has demonstrated how *Enterococcus* have consistently been the second most isolated urinary bacteria, after *E. coli*, from catheterized ICU patients, irrespective of decade (1990–2007) and symptomatology (14). The European Centre for Disease Prevention and Control (ECDC) did in two surveillance reports, years 2008–2012 and 2017, show that *Enterococcus* was the second most reported ICU-based UTI in the EU as well, again only following *E. coli* (15, 16). Unlike numerous EU members, Sweden has not adopted the definitions suggested by the ECDC, leading to absence in global statistics (2, 15–17). Regional healthcare instead recognizes clinical definitions of UTIs, characterized by diagnostics on symptomatology, urine dipsticks and C-reactive protein levels, occasionally aided by medical imaging (18). Urine cultivation is only performed for species determination and AMR-profiling on clinical indication of a UTI, and thresholds for significance (colony forming units) is determined by factors such as disease severity, sampling local, sampling method and bacterial pathogenicity group (19, 20). While practical definitions might suffice in treating individual patients, the absence of a definition can confine on equal care and antibiotic stewardship programs, while also preventing comparative representation (18). Swedish authorities have asserted that results from occasional national surveillance have been in accordance with neighboring European countries (17). Local authorities did in 2019 however, recognize UTIs as the main cause of Swedish HAIs, accounting for a staggering 60.8%, founded on marker-based journal-evaluations (2). The same report estimated the direct mortality of nosocomial UTIs to 0.4%, but indirect mortality to 4.8% (mainly due to secondary sepsis), and also showed that only having a UTI compared to not having any HAI increased inpatient care with 7.5 days. The estimated cost for Swedish inpatients corresponds to approx. 1000 EUR/day, thus putting the projected additional cost per UTI-patient to 7500 EUR.

Our study found few *E. coli*, but also few *P. aeruginosa* and a complete absence of other common Gram-negative UTI pathogens, such as *Klebsiella* and *Proteus* in urine. Immunosuppressives are known to increase risk for infection, and while the β-lactam-heavy treatment might have prevented most Gram-negative bacteria from colonizing, the regimen has little effect on more tolerant Gram-positives. Internal cephalosporin-carbapenem tolerance among *Enterococcus* spp. is well-described (21), and *in vitro* MIC results confirmed that all *E. faecium* isolates were penicillin-aminoglycoside tolerant (Figure 4). While the overall antibiotic use went down in the ICU compared to 2019 (pre-pandemic), we illustrate here how the specific use of meropenem, and particularly cefotaxime, radically increased in 2020 (Figure 5C). Moreover, we demonstrate how the prevalence of *Enterococcus* in urine coincided with treatment of meropenem and cefotaxime (Figures 5A, B). These factors considered; we can conclude that the high use of these β-lactams likely contributed to the proportionally elevated prevalence of *Enterococcus* in the ICU.

*E. coli* bacteriuria mainly occurred in concurrence with Gram-positive colonizers. While it would be intriguing to suggest that drug resistant *Enterococcus* might protect susceptible *E. coli*, our study suggests a more complex picture (Supplementary Figure 3). Two *E. coli* were separately isolated with MDR *E. faecium* and *S. epidermidis*, but three isolates were found alongside antibiotic susceptible *E. faecalis*. Antibiotic susceptibility in *E. faecalis* did however, not affect the strains’ ability to colonize/survive. Apparent from our timeline of patient B was the agreement between *in vitro* susceptibility against TZP, and bacteriuria clearance of both *E. faecalis* and co-colonizing *E. coli* (Figure 6B). Importantly, the same clonal *E. faecalis* (confirmed by WGS) reappeared a full 12 days later, only to be followed by the same clonal *E. coli*. Previous molecular studies *in vitro* have shown how *Enterococcus* promote infection of *E. coli* through biofilm formation, increased virulence, and suppression of the immune system (22–24). Cases reporting *Enterococcus* spp. preceding *E. coli in vivo* are rare. Uropathogens in biofilms are known to endure with minimal metabolic activity, especially on urinary catheters. Virulent UTI bacteria, such as UPEC, have moreover been shown to adhere in extracellular matrix, inside cells, or deeper tissue layers (25). *Enterococcu*s is known to act as a pioneer-species for polymicrobial colonization of catheters *in vitro* (26), and importantly in the case of patient B, the catheter had not been changed. When the catheter later was exchanged in combination with cefotaxime, *E. coli* was cleared and *E. faecalis* demonstrated a 10^3^ CFU/ml-drop. The *E. coli* had *in vitro* susceptibility but *E. faecalis* are intrinsically resistant while still experiencing the CFU-reduction, suggesting biofilm on the catheter (Figure 6B). Only 1/15 *Enterococcus* isolates started appearing before 7 days of catheterization, signifying that adequate time is needed to establish colonization (Supplementary Table 1). Virulence and persistence mechanisms for these strains would require further genetic and molecular investigations out of scope for the present study.

Isolates from patient A demonstrated a MIC-increase against PIP and TZP during antibiotic treatment (Figure 6A). Rapid changes in AMR have previously been explained by heteroresistance (27), phase variation, gene amplification, plasmid copy number increase, or epigenetic modifications (28). Notably, EUCAST and the Clinical Laboratory Standards Institute (CLSI) indicate ampicillin for inferred resistance against PIP, yet our strains of *E. faecium* demonstrated PIP-TZP and imipenem resistance while being ampicillin-susceptible. This has previously been reported in *E. faecalis*, but to the best of our knowledge not in *E. faecium* (29, 30). That the addition of tazobactam escalates the MIC against PIP is concerning, especially given the broad use of this combination. Molecular studies are needed to elucidate underlying mechanisms for resistance in these strains.

Our study brings attention to several limitations when assessing nosocomial UTIs. Not having a global consensus when defining these infections converts a concern when reviewing previous studies and meta reports, where a discord in significance thresholds and distinguishment between diagnosis and microbial findings make comparative conclusions challenging. In concord with previous studies, we too want to highlight the risk for hidden UTI statistics during systemic inflammation and kidney injury, where primary diagnostic criteria might be masked (7, 31). We also recognize that our study has limitations. Our investigation did not allow for follow-up on colonization and treatment before or after ICU stay, hence we cannot rule out pre/post ICU antibiotics and bacteriuria. Cultivation did not allow for detection of anaerobic bacteria and might have reduced transient gene- or plasmid amplification events in relation to antibiotic resistance. As our permit allowed for non-invasive sample collection, we could not assess microbial growth in patients experiencing anuria.

In conclusion, we identified LOS as a predisposing factor for bacteriuria in Swedish COVID-19 ICU patients. We detected MDR bacteria defined as “critical” or of high concern on the WHO priority list (32, 33). High-level use of β-lactams, especially cefotaxime, likely contributed to a disproportionally high prevalence of Gram-positive colonizers and MDR bacteria, mostly *Enterococcus*. The ability of *E. coli* to cause bacteriuria despite effective antibiotic treatment, when found in co-culture with cephalosporin-tolerant *Enterococcus*, highlights the role of biofilm in urinary catheters as a reservoir of pathogenic bacteria with the potential to develop and disseminate AMR. We want to stress that AMR and healthcare-associated UTIs increase healthcare costs and constitute persistent risks for patients, and that polymicrobial biofilms in catheters probably are more common and complicated than what the categories of UTI diagnostics might imply. This study provides new insight into the role of ICU stay and antibiotic use in shaping bacteriuria, and how colonization permits polymicrobial communities of susceptible but pathogenic bacteria to remain during treatment.

## Data availability statement

The original contributions presented in this study are included in this article/Supplementary material, further inquiries can be directed to the corresponding authors.

## Ethics statement

Data found in this research are part of the PronMed study approved by the National Ethical Review Agency [Dnr 2017/043 (with amendments 2020-01623, 2020-02719, 2020-05730, and 2021-01469) and 2022-00526-01)] and listed at ClinicalTrials.gov (NCT03720860). Informed consent was obtained from the patient or next of kin. The Declaration of Helsinki and its subsequent revisions were followed. Adult ICU patients admitted between the June 5, 2020 and February 17, 2021 with reverse-transcription polymerase chain reaction (RT-PCR) positive nasopharyngeal swabs were prospectively recruited to the study. Exclusion criteria were pregnancy, current breastfeeding, and age under 18. End of follow-up was defined to the end of ICU treatment.

## Author contributions

PK, HW, and JJ: conceptualization and writing—original draft. PK, ED, JP, MH, and RF: data curation. PK, JP, NF-K, HW, and JJ: formal analysis. HW, MH, RF, and JJ: funding acquisition. PK, JP, HW, and JJ: investigation. PK, HW, JJ, MH, RF, and ML: methodology. HW, JJ, MH, and RF: project administration. HW and JJ: validation. JP, ED, NF-K, MH, RF, and ML: writing—review and editing. All authors contributed to the interpretation of results and critical review of the manuscript and had access to the data, except for identifiable clinical data to which access was restricted to those acquiring and analyzing it.
