# Supplementary material for: Antibiotic use during coronavirus disease 2019 intensive care unit shape multidrug resistance bacteriuria: A Swedish longitudinal prospective study
Source: Front Med (Lausanne). 2023 Feb 7;10:1087446. doi: 10.3389/fmed.2023.1087446 (PMC9941185; doi:10.3389/fmed.2023.1087446)
Supplement: Supplementary file 1 [file Data_Sheet_1.DOCX]

**Supplementary Material**

**Species identification and cultivation**

To isolate bacterial colonies, 100 µl thawed urine (diluted 1:2 in PBS) was streaked onto Brilliance™ UTI Clarity™ agar (chromogenic agar, CM1106 Oxoid) plates, incubated at 37°C for 24h according to the manufacturer’s instructions. To avoid bottlenecking genotypes, ten colonies (if accessible) per indicated species were picked in the same loop and streaked onto new agar plates to purify cultures. The cutoff for significant growth was in this study considered >10^3^ CFU/ml (>10^5^ for *Staphylococcus epidermidis*) with >50 countable colonies per 50 µl urine (see supplementary table 1 for more information). Bacterial species were identified using matrix-assisted laser desorption/ionization time-of-flight mass spectrometry (MALDI-TOF, Bruker Biotyper^®^), and dissolved colonies were frozen in Brain Heart Infusion (BHI) broth with 10% DMSO. Species confirmation and clonality control was performed for a subset of strains with whole-genome sequencing analysis and included long-scaffold BLAST, 16S analysis (SpeciesFinder 2.0)(1), MLST-typing (pubMLST), serotyping (*Escherichia* & *Pseudomonas*)(2,3), and spa-typing (*Staphylococcus*)(4)*.* Sequence control and assembly were performed in CLC Genomics Workbench v21 as previously described from our lab (5,6). For sequencing, *Escherichia, Pseudomonas,* and *Staphylococcus* were grown with aeration on Luria agar (LA) and in Luria broth (LB) with shaking. *Enterococcus* were grown on BHI agar and in BHI broth with shaking, *E. faecalis* aerobically, and *E. faecium* in microaerophilic tanks with atmosphere-altering kits (Oxoid™ CampyGen™ 2.5L, ThermoFisher). In preparation for antimicrobial susceptibility testing, all strains were grown aerobically on Mueller Hinton (MH) agar plates, 24h for all strains except *Enterococcus* which required 48h. All cultivation was carried out at 37°C.

**Supplementary Table S1. Information on microbiological thresholds.**

No global consensus exists in the definition of microbiological significance for urinary tract infections. General agreements are found in that clinical symptoms should preferably coincide with growth of bacteria in urine (at a certain threshold), confirmed via colony forming unit count, or microscopy-related assessment. Different countries and institutions might or might not take catheterization or bacterial pathogenicity into account when defining UTIs.

Swedish healthcare performs UTI diagnoses on symptomatology, chemical rapid tests (nitrate and leucocytes) and C-reactive protein level, occasionally aided by medical imaging (ultrasound/DMSA scintigraphy & bladder X-ray). Routine urine cultivation is made semiquantitatively for species determination and AMR profiling on indication of UTI, and on treatment failure. A diagnosis for healthcare-associated UTIs does currently not exist in Sweden, but catheter-associated UTI is still practically distinguished from non-catheterized UTI with regards to CFU threshold levels. This is different from the American CDC and the European ECDC which both use >10^5^ CFU/ml as a threshold for all bacteria and collection methods (7). The Swedish Medical Products Agency has defined microbiological thresholds as following:

1. Primary pathogens (midstream urine): >10^3^ CFU/ml
2. Secondary pathogens (midstream urine): >10^4^ CFU/ml
3. Doubtful pathogens (midstream urine): >10^5^ CFU/ml
4. Any bacteria (bladder puncture): >10^2^ CFU/ml
5. Any bacteria while UTI symptoms (from indwelling catheter): >10^3^ CFU/ml

Clinical symptoms involved in assessment of UTIs include local pain and miction dysfunction (acute cystitis), fever and occasional abdominal pain (febrile UTI). All symptoms are arguably concealed by sedation or primary COVID-19 (8). The table below illustrates if the isolate is considered significant by Swedish and ECDC standards, and if the isolate was identified by routine microbiology.

| Isolate | Species | Sweden | ECDC | Routine Finding | ICU day: 1st isolate |
| --- | --- | --- | --- | --- | --- |
| 1 | *Morganella morganii* | Yes | No | - | 10 |
| 2 | *Escherichia coli* | Yes | No | Yes | 10 |
| 3 | *Enterococcus faecalis* | Yes | No | - | 10 |
| 4 | *Enterococcus durans* | Yes | No | - | 22 |
| 5 | *Enterococcus faecium* | Yes | Yes | - | 24 |
| 6 | *Escherichia coli* | Yes | Yes | Yes | 24 |
| 7 | *Enterococcus faecium* | Yes | No | Yes | 7 |
| 8 | *Enterococcus durans* | Yes | No | - | 19 |
| 9 | *Pseudomonas aeruginosa* | Yes | Yes | - | 25 |
| 10 | *Enterococcus faecalis* | Yes | Yes | - | 32 |
| 11 | *Staphylococcus aureus* | Yes | Yes | - | 32 |
| 12 | *Corynebacterium* | Yes | No | - | 2 |
| 13 | *Escherichia coli* | Yes | Yes | Yes | 2 |
| 14 | *Enterococcus faecalis* | Yes | Yes | - | 19 |
| 15 | *Escherichia coli* | Yes | No | Yes | 19 |
| 16 | *Enterococcus faecium* | Yes | Yes | Yes | 18 |
| 17 | *Staphylococcus hominis* | Yes | Yes | - | 3 |
| 18 | *Escherichia coli* | Yes | No | - | 3 |
| 19 | *Enterococcus faecalis* | Yes | No | - | 3 |
| 20 | *Pseudomonas aeruginosa* | Yes | Yes | Yes | 10 |
| 21 | *Staphylococcus capitis* | Yes | Yes | - | 2 |
| 22 | *Staphylococcus epidermidis* | Yes | No | - | 9 |
| 23 | *Citrobacter koseri* | Yes | Yes | - | 33 |
| 24 | *Citrobacter koseri* | Yes | No | - | 43 |
| 25 | *Enterococcus faecium* | Yes | No | - | 19 |
| 26 | *Enterococcus faecalis* | Yes | Yes | - | 33 |
| 27 | *Enterococcus faecium* | Yes | No | - | 38 |
| 28 | *Enterococcus faecium* | Yes | Yes | Yes | 33 |
| 29 | *Staphylococcus epidermidis* | Yes | Yes | - | 4 |
| 30 | *Escherichia coli* | Yes | Yes | Yes | 4 |
| 31 | *Enterococcus faecium* | Yes | Yes | - | 9 |
| 32 | *Corynebacterium* | Yes | No | - | 2 |
| 33 | *Staphylococcus haemolyticus* | Yes | No | - | 2 |
| 34 | *Enterococcus faecalis* | Yes | Yes | Yes | 8 |

**

**

**Supplementary Figure S1. A-C.** Automatic binning (GraphPad) of continuous parameters by frequency distribution. Values for significance is calculated by the D'Agostino-Pearson test for normal distribution and Chi-squared test for length of stay (LOS) grouping (only C). **D.** Cross-correlation analysis of age, death, LOS, and simplified acute physiology score 3 (SAPS3), where 1.0 is 100% correlation (dark blue), 0.0 is 0% correlation (white) and -1.0 is 100% inverse correlation (red). Values for self-correlation have been removed and the p-values are given within brackets.


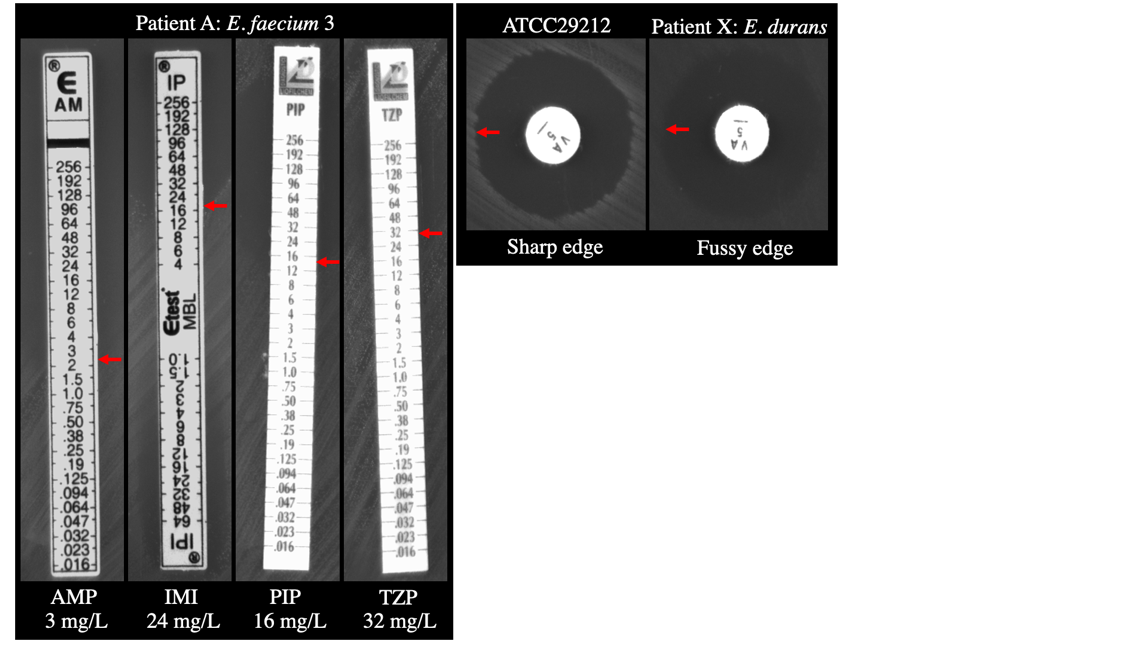


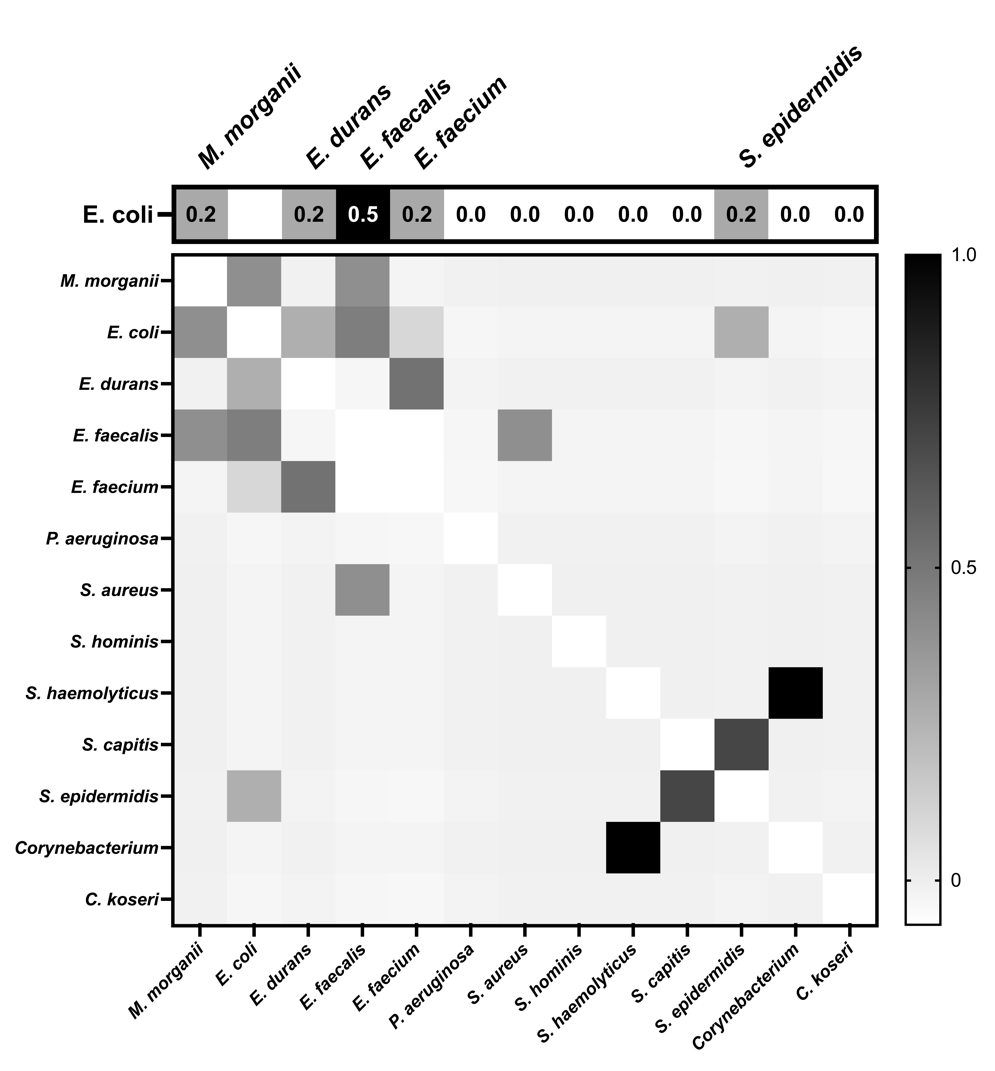
**Supplementary Figure S2.** Examples from confirmatory tests for *E. faecium* showing AMP-susceptibility during IMI-PIP-TZP resistance, and higher TZP MIC compared to PIP. Disk diffusion for *E. durans* show fussy edges as compared to the reference sharp edges, as an indication for VAN resistance.

**Supplementary Figure S3.** Heatmap matrix showing the proportional correlation of urine co-colonizers ranging from 0% (light grey) to 100% (black), self-correlation has been removed from analysis (white). A separate species-based analysis is indicated with an additional bar for *E. coli* to highlight proportional co-colonization within the species.

**Supplementary Table S2. Information on tested antibiotics from EUCAST.**

Strains with an identified MIC between “R” and “S” were before 2019 classified as intermediates (“I”). Since 2019, this previously intermediate group is now referred to as “susceptible, increased exposure”, still designated with the letter “I” in the SIR classification. This means that bacteria can only group in “I” if the antibiotic tested can be administered at an increased dose. Internal validation was performed with EUCAST-recommended type strains *E. faecalis* ATCC 29212, *E. coli* ATCC 25922 and *P. aeruginosa* ATCC 27853 with both BMD and E-tests. MRSA strain *S. aureus* ATCC 33591 was additionally included for validation of cefoxitin-resistance, *K. quasipneumoniae* ATCC 700603 for validation of beta-lactam/beta-lactamase resistance and *E. faecalis* ATCC 51299 when confirming HLAR/VanB resistance. Vancomycin for *Enterococcus* and cefoxitin for *Staphylococcus* were in addition to BMD moreover controlled with the disk diffusion method as recommended by EUCAST. Tigecycline resistance was additionally confirmed with E-tests due to the importance of fresh media. Results were only accepted if controls were functional, and the duplicates did not deviate more than one dilution. Antibiotics used are listed below. Tazobactam was added as 4mg/L after dilution of piperacillin and trimethoprim was added in a 1:19 ratio with sulfamethoxazole. Discrepancies were always confirmed with E-tests.

|  | *Enterobacterales* | *Pseudomonas* | *Staphylococcus* | *Corynebacterium* | *Enterococcus* |
| --- | --- | --- | --- | --- | --- |
| Trimethoprim (TMP) | >4 mg/L | NA | >4 mg/L | NA | NA |
|  | If not resistant: susceptible | - | If not resistant: susceptible | - | - |
| Trimethoprim-Sulfamethoxazole (SXT) | >4 mg/L | NA | >4 mg/L | NA | NA |
|  | If >2 mg/L: susceptible, increased exposure. Added in 1:19 ratio trimethoprim: sulfamethoxazole | - | If >2 mg/L: susceptible, increased exposure. Added in 1:19 ratio trimethoprim: sulfamethoxazole | - | - |
| Cefotaxime (CTX) | >2 mg/L | NA | NA | NA | NA |
|  | If >1 mg/L: susceptible, increased exposure. | - | Susceptibility inferred from cefoxitin | - | - |
| Gentamicin  (GEN) | >2 mg/L | NA | >2 mg/L | NA | >128 mg/L |
|  | If not resistant: susceptible | - | If not resistant: susceptible | - | Ineffective on its own, but synergy with beta-lactams when not expressing high-level aminoglycoside resistance (HLAR). If >128 mg/L: HLAR-type against GEN and all other aminoglycosides but streptomycin. Negative HLAR might not mean negative for other aminoglycosides. |
| Tobramycin  (TOB) | >2 mg/L | >2 mg/L | >2 mg/L | NA | >128 mg/L |
|  | If not resistant: susceptible | If not resistant: susceptible | If not resistant: susceptible | - | Ineffective on its own, but synergy with beta-lactams when not expressing high-level aminoglycoside resistance (HLAR). If >128 mg/L: HLAR-type against TOB. Negative HLAR might not mean negative for other aminoglycosides. |
| Ampicillin  (AMP) | >8 mg/L | NA | NA | NA | >8 mg/L |
|  | If not resistant: susceptible | - | Susceptibility inferred from penicillin G, only used for *S. saprophyticus* | - | If >4 mg/L: susceptible, increased exposure. Ampicillin is used to infer resistance against amoxicillin and piperacillin (with and without lactamase inhibitors). Ampicillin resistance is common in *E. faecium* but not in *E. faecalis*. |
| Piperacillin (PIP) | >8 mg/L | >16 mg/L | NA | NA | NA |
|  | If not resistant: susceptible | If >0.001 mg/L: susceptible, increased exposure. | Susceptibility inferred from penicillin G | - | Susceptibility inferred from ampicillin. |
| Piperacillin-Tazobactam (TZP) | >8 mg/L | >16 mg/L | NA | NA | NA |
|  | If not resistant: susceptible. Tazobactam fixed at 4 mg/L, MIC measured in [piperacillin]. | If >0.001 mg/L: susceptible, increased exposure. Tazobactam fixed at 4 mg/L, MIC measured in [piperacillin]. | Susceptibility inferred from cefoxitin. Strains resistant to penicillin G but susceptible to cefoxitin are susceptible to lactamase inhibitors | - | Susceptibility inferred from ampicillin. |
| Meropenem (MEM) | >8 mg/L | >8 mg/L | NA | NA | NA |
|  | If >2 mg/L: susceptible, increased exposure. | If >2 mg/L: susceptible, increased exposure. | Susceptibility inferred from cefoxitin. | - | - |
| Imipenem (IMI) | >4 mg/L | >4 mg/L | NA | NA | >4 mg/L |
|  | If >2 mg/L: susceptible, increased exposure. For *M. morganii*, *Proteus* spp. and *Providencia* spp., if >0.001 mg/L: susceptible, increased exposure. | If >0.001 mg/L: susceptible, increased exposure. | Susceptibility inferred from cefoxitin. | - | If >0.001 mg/L: susceptible, increased exposure. |
| Tigecycline  (TGC) | >0.5 mg/L | NA | >0.5 mg/L | NA | >0.25 mg/L |
|  | If not resistant: susceptible. For BMD, MH media must be prepared fresh on day of use. TGC activity insufficient against *M. morganii*, *Proteus* spp. and *Providencia* spp. | - | If not resistant: susceptible. For BMD, MH media must be prepared fresh on day of use. Resistant isolates are rare or not yet reported. | - | If not resistant: susceptible. For BMD, MH media must be prepared fresh on day of use. Resistant isolates are rare or not yet reported. |
| Ciprofloxacin  (CIP) | >0.5 mg/L | >0.5 mg/L | >1 mg/L | >1 mg/L | >4 mg/L |
|  | If >0.25 mg/L: susceptible, increased exposure. | If >0.001 mg/L: susceptible, increased exposure. | If >0.001 mg/L: susceptible, increased exposure. | If >0.001 mg/L: susceptible, increased exposure. | If not resistant: susceptible. |
| Aztreonam (ATM) | >4 mg/L | >16 mg/L | NA | NA | NA |
|  | If >1 mg/L: susceptible, increased exposure. | If >0.001 mg/L: susceptible, increased exposure. | - | - | - |
| Ceftazidime (CAZ) | >4 mg/L | >8 mg/L | NA | NA | NA |
|  | If >1 mg/L: susceptible, increased exposure. | If >0.001 mg/L: susceptible, increased exposure. | - | - | - |
| Vancomycin  (VAN) | NA | NA | >4 mg/L (>2 mg/L) | >2 mg/L | >4 mg/L |
|  | - | - | If not resistant: susceptible. Resistance rare or not yet reported. If *S. aureus*: resistance at >2 mg/L, if at exactly 2 mg/L: potential impaired clinical response. | If not resistant: susceptible. | If not resistant: susceptible. Recommended to test with disk diffusion (<12 mm = resistant). Examine in transmitted light, fuzzy edges are resistant even if diameter is >12 mm. Disk diffusion must be run at least 24 hours. |
| Clindamycin (CLI) | NA | NA | >0.25 mg/L | >0.5 mg/L | NA |
|  | - | - | If not resistant: susceptible. | If not resistant: susceptible. | - |
| Linezolid (LZD) | NA | NA | >4 mg/L | >2 mg/L | >4 mg/L |
|  | - | - | If not resistant: susceptible. | If not resistant: susceptible. | If not resistant: susceptible. |
| Penicillin G /benzylpenicillin  (BENPEN) | NA | NA | >0.125 mg/L | >0.125 mg/L | NA |
|  | - | - | If not resistant: susceptible. Isolates that test resistant to penicillin G in *S. aureus* and *S. lugdunensis* are penicillinase producers and resistant to all penicillins. No reliable detection of penicillinases in other staphylococci currently exist. | If not resistant: susceptible. | - |
| Cefoxitin (FOX) | NA | NA | >4 mg/L  (>8 mg/L) | NA | NA |
|  | - | - | If not resistant: susceptible. All relevant beta-lactam resistance can be inferred with FOX (also methicillin). *S. aureus* and *S. lugdunensis* are resistant when >4 mg/L, *S. saprophyticus* when >8 mg/L. When screening for methicillin resistance, disk diffusion best predicts resistance. | - | - |
| Erythromycin (ERY) | NA | NA | >2 mg/L | NA | NA |
|  | - | - | If >1 mg/L: susceptible, increased exposure. Strains susceptible to ERY are also susceptible to AZM, clarithromycin and roxithromycin. ERY-resistant strains should be tested for susceptibility to individual reagent. | - | - |
| Azithromycin (AZM) | NA | NA | >2 mg/L | NA | NA |
|  | - | - | If not resistant: susceptible. | - | - |

**Supplementary References**

1. Larsen M V., Cosentino S, Lukjancenko O, Saputra D, Rasmussen S, Hasman H, et al. Benchmarking of methods for genomic taxonomy. J Clin Microbiol [Internet]. 2014 [cited 2022 May 30];52(5):1529–39. Available from: https://pubmed.ncbi.nlm.nih.gov/24574292/

2. Thrane SW, Taylor VL, Lund O, Lam JS, Jelsbak L. Application of Whole-Genome Sequencing Data for O-Specific Antigen Analysis and In Silico Serotyping of Pseudomonas aeruginosa Isolates. J Clin Microbiol [Internet]. 2016 Jul 1 [cited 2022 May 30];54(7):1782–8. Available from: https://pubmed.ncbi.nlm.nih.gov/27098958/

3. Joensen KG, Tetzschner AMM, Iguchi A, Aarestrup FM, Scheutz F. Rapid and Easy In Silico Serotyping of Escherichia coli Isolates by Use of Whole-Genome Sequencing Data. J Clin Microbiol [Internet]. 2015 Aug 1 [cited 2022 May 30];53(8):2410–26. Available from: https://pubmed.ncbi.nlm.nih.gov/25972421/

4. Bartels MD, Petersen A, Worning P, Nielsen JB, Larner-Svensson H, Johansen HK, et al. Comparing Whole-Genome Sequencing with Sanger Sequencing for spa Typing of Methicillin-Resistant Staphylococcus aureus. J Clin Microbiol [Internet]. 2014 Dec 1 [cited 2022 May 30];52(12):4305. Available from: /pmc/articles/PMC4313303/

5. Karlsson PA, Tano E, Jernberg C, Hickman RA, Guy L, Järhult JD, et al. Molecular Characterization of Multidrug-Resistant Yersinia enterocolitica From Foodborne Outbreaks in Sweden. Front Microbiol [Internet]. 2021 May 13 [cited 2022 May 30];12. Available from: https://pubmed.ncbi.nlm.nih.gov/34054769/

6. Karlsson PA, Hechard T, Jernberg C, Wang H. Complete Genome Assembly of Multidrug-Resistant Yersinia enterocolitica Y72, Isolated in Sweden. Microbiol Resour Announc [Internet]. 2021 May 13 [cited 2022 May 30];10(19). Available from: https://pubmed.ncbi.nlm.nih.gov/33986087/

7. European centre for disease prevention and control. Surveillance of healthcare-associated infections and prevention indicators in European intensive care units - HAI-Net ICU protocol version 2.2. Eur Cent Dis Prev Control [Internet]. 2017 [cited 2022 Sep 13];1–50. Available from: www.ecdc.europa.eu

8. Läkemedelsverket. Läkemedelsbehandling av urinvägsinfektioner i öppenvård – behandlingsrekommendation. In: Information från Läkemedelsverket nr 5 2017 [Internet]. Uppsala: Läkemedelsverket; 2017 [cited 2022 Sep 13]. p. 16. Available from: https://www.lakemedelsverket.se
